# The path to visualization of walking myosin V by high-speed atomic force microscopy

**DOI:** 10.1007/s12551-014-0141-7

**Published:** 2014-06-18

**Authors:** Noriyuki Kodera, Toshio Ando

**Affiliations:** 1 Bio-AFM Frontier Research Center, Kanazawa University, Kanazawa, 920-1192 Japan; 2 Department of Physics, College of Science and Engineering, Kanazawa University, Kakuma-machi, Kanazawa, 920-1192 Japan; 3 PREST, Japan Science and Technology Agency, 4-1-8 Honcho, Kawaguchi, 332-0012 Japan; 4 CREST, Japan Science and Technology Agency, 4-1-8 Honcho, Kawaguchi, 332-0012 Japan

**Keywords:** Myosin, Actin, Muscle, Motor proteins, High-speed AFM, Imaging

## Abstract

The quest for understanding the mechanism of myosin-based motility started with studies on muscle contraction. From numerous studies, the basic frameworks for this mechanism were constructed and brilliant hypotheses were put forward. However, the argument about the most crucial issue of how the actin–myosin interaction generates contractile force and shortening has not been definitive. To increase the “directness of measurement”, in vitro motility assays and single-molecule optical techniques were created and used. Consequently, detailed knowledge of the motility of muscle myosin evolved, which resulted in provoking more arguments to a higher level. In parallel with technical progress, advances in cell biology led to the discovery of many classes of myosins. Myosin V was discovered to be a processive motor, unlike myosin II. The processivity reduced experimental difficulties because it allowed continuous tracing of the motor action of single myosin V molecules. Extensive studies of myosin V were expected to resolve arguments and build a consensus but did not necessarily do so. The directness of measurement was further enhanced by the recent advent of high-speed atomic force microscopy capable of directly visualizing biological molecules in action at high spatiotemporal resolution. This microscopy clearly visualized myosin V molecules walking on actin filaments and at last provided irrefutable evidence for the swinging lever-arm motion propelling the molecules. However, a peculiar foot stomp behavior also appeared in the AFM movie, raising new questions of the chemo-mechanical coupling in this motor and myosin motors in general. This article reviews these changes in the research of myosin motility and proposes new ideas to resolve the newly raised questions.

## Introduction

Motions in biological systems are vital phenomena. They inspire our curiosity to understand how motion is made possible. Skeletal muscles exhibit striking physiological action (i.e. contraction) that is visible to the naked eye and is daily experienced by ourselves. It was, therefore, natural that research into biological motility started with the analysis of muscles. Biochemical studies of muscle proteins revealed that the molecular entities responsible for generating contractile force and shortening comprise just three: myosin, actin, and ATP. Each head of the double-headed myosin hydrolyzes ATP into ADP and inorganic phosphate (Pi). The ATPase rate is very low when myosin is alone but is markedly accelerated by its interaction with actin, where the chemical energy liberated by ATP hydrolysis is converted into mechanical work. This energy conversion has most attracted our curiosity and thus been extensively studied using physiological, biophysical, biochemical, structural biological, and theoretical methods. It is noteworthy that several experimental techniques have been applied or created to study muscle contraction, and later used in other fields of biological science. Results obtained in each discipline have built important frameworks and hypotheses for the mechanism of muscle contraction. For example: (1) the relationship between load, speed of shortening, and the rate of heat production in muscle contraction (Hill 1938, 1964); (2) the filament sliding mechanism in which muscle shortens as a result of the mutual sliding of thin filaments containing actin relative to thick filaments containing myosin (Huxley 1953; Huxley and Hanson 1954; Huxley and Niedergerke 1954); (3) the generation of force by myosin cross-bridges that extend from the myosin filaments to the adjacent actin filaments in the overlap zone in the sarcomere (Huxley 1957a, b); (4) the hypothesis of swinging lever-arm as powerstroke (Huxley 1969); (5) the concept of a working stroke of ∼10 nm derived from the swinging lever-arm hypothesis and from the observation of tension recovery following a quick release applied to contracting muscle fibers (Huxley and Simmons 1971); and (6) the kinetic scheme of the actomyosin ATPase reaction (Lymn and Taylor 1971). These ideas were integrated into a general consensus on the muscle contraction mechanism. Nonetheless, a clue to greater clarification of the chemo-mechanical reaction in actomyosin unexpectedly emerged from the discovery of non-muscle myosins together with the creation of single-molecule biophysical techniques.

Currently, there are 35 (or more) distinct classes of myosins designated I to XXXV (Berg et al. 2001; Thompson and Langford 2002; Richards and Cavalier-Smith 2005; Foth et al. 2006; Odronitz and Kollmar 2007). They constitute the superfamily of actin-based motors that play crucial roles in dynamic cellular processes. In the initial attempt to find non-muscle myosins, first a single-headed myosin (Pollard and Korn 1973) and subsequently a traditional double-headed myosin (Maruta and Korn 1977) were isolated from *Acanthamoeba*. Later, the former was called “myosin I” and the latter “myosin II”. Thereafter, the Roman numerals have been given according to the chronological order of discovery. Among the 35 classes of myosins, myosin V (Mehta et al. 1999; Sakamoto et al. 2000), VI (Rock et al. 2001; Nishikawa et al. 2002), VII (Yang et al. 2006), X (Nagy et al. 2008; Ricca and Rock 2010; Sun et al. 2010), and XI (Tominaga et al. 2003; Hachikubo et al. 2007) are known to be processive motors, i.e. a single molecule undergoes multiple catalytic cycles and mechanical advances before dissociation from an actin filament. This processivity promoted the study of myosin motility because it enabled us to continuously trace the motor action of individual molecules. Note that not all isoforms in each of these classes are processive. For example, the yeast myosin V, Myo2p (Reck-Peterson et al. 2001), and the *Chara* myosin XI (Kimura et al. 2003) are not processive, although Myo2p is switched to a processive motor when tropomyosin is bound to actin filaments (Hodges et al. 2012).

In parallel with the progress of non-muscle myosin studies, fluorescence microscopy (see review by Joo et al. 2008) and optical trap nanometry (see review by Neuman and Block 2004) were developed to observe the dynamic action of proteins at the single molecule level. These techniques assisted the discovery of several properties of myosin V (hereafter referred to as M5) including three-to-five successive step advances under load with the ∼36-nm step size (Mehta et al. 1999), processive runs of several micrometers under no load (Sakamoto et al. 2000), load-dependent stepping kinetics (Veigel et al. 2002), the hand-over-hand manner of this movement (Forkey et al. 2003; Yildiz et al. 2003), and the tight relationship between the catalytic cycle and the 36-nm advance (Sakamoto et al. 2008). These discoveries led to a comprehensive view for the action of myosin motors. Nonetheless, a complete consensus has not been reached and more details of the motor action and the underlying chemo-mechanical coupling have remained elusive.

Looking back on the long history of myosin motility studies, we notice that the gap between experimental data and conclusions derived therefrom has been slowly reducing. This is because the level of “directness of measurement” has been increasing. Before the single-molecule era, we had to infer the action of individual molecules from experimental data ensemble-averaged over many molecules. Several details of the action of myosin were hidden in the ensemble-average measurements on bulk samples. However, even when single-molecules were observed, their level of directness was not perfect because the subset of observed molecular events depends on the site where an optical probe is placed while the entire molecular structure remained invisible. Myosin has been studied by X-ray crystallography, NMR, and electron microscopy, but these structures are limited to static snapshots and the simultaneous assessment of structure and dynamics seemed impossible.

High-speed atomic force microscopy (HS-AFM), which started to be developed around 1993 to overcome this limitation, is now sufficiently advanced (Ando et al. 2008) to allow us to video-record the structure and dynamics of functioning biomolecules at single-nanometer resolution, without disturbing their function. This new microscopy was applied to M5 (Kodera et al. 2010). The obtained image data revealed details of its molecular action, leading not only to the corroboration of known or inferred behavior of the motor but also to new discoveries (Kodera et al. 2010). In particular, it was a surprise to discover that the tension responsible for forward movement can be generated without transitioning through an ADP–Pi-bound state, meaning that no chemical energy input is required for the tension generation. Moreover, the lever-arm swing (powerstroke) by the leading head spontaneously occurs when the trailing head detaches, thus demonstrating that no chemical energy input is required for the lever-arm swing either. These findings appear inconsistent with a popular scheme put forward for the chemo-mechanical reaction in muscle contraction (e.g., Geeves and Holmes 1999; Goldman 2004). Thus, we now need to develop a unified motor mechanism that can be applied to all classes of myosin motors.

This review does not cover the entire range of myosin motors but focuses on studies of the motor mechanism, particularly those performed with the use of single-molecule techniques and our recent HS-AFM study of M5 motility. Readers are referred to reviews on muscle contraction (Geeves and Holmes 1999; Huxley 2000, 2004; Goldman 2004), on structure–function relationships of myosins (Ruppel and Spudich 1996; Sweeney and Houdusse 2010a), and on the cellular functions of various myosins (Hartman et al. 2011; Coluccio 2008).

## General aspects of myosin

**Fig. 1:**
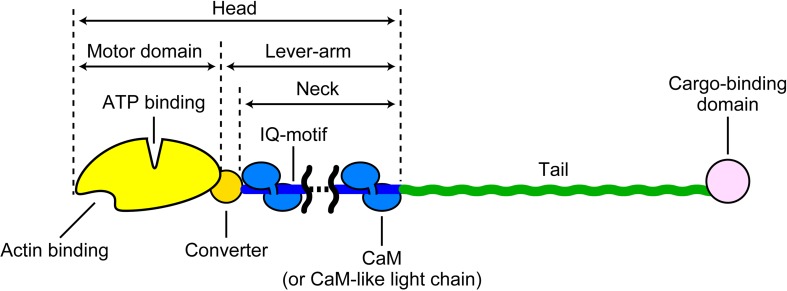
Schematic showing domain organization of myosin molecule. The tail part is variable and contains an α-helical coiled-coil, a SAH domain, or a chain segment with a variable amino-acid sequence, depending on the myosin class or isoform. When a SAH domain follows the neck, it is included in the lever-arm because it functionally extends the canonical lever-arm

**Fig. 2:**
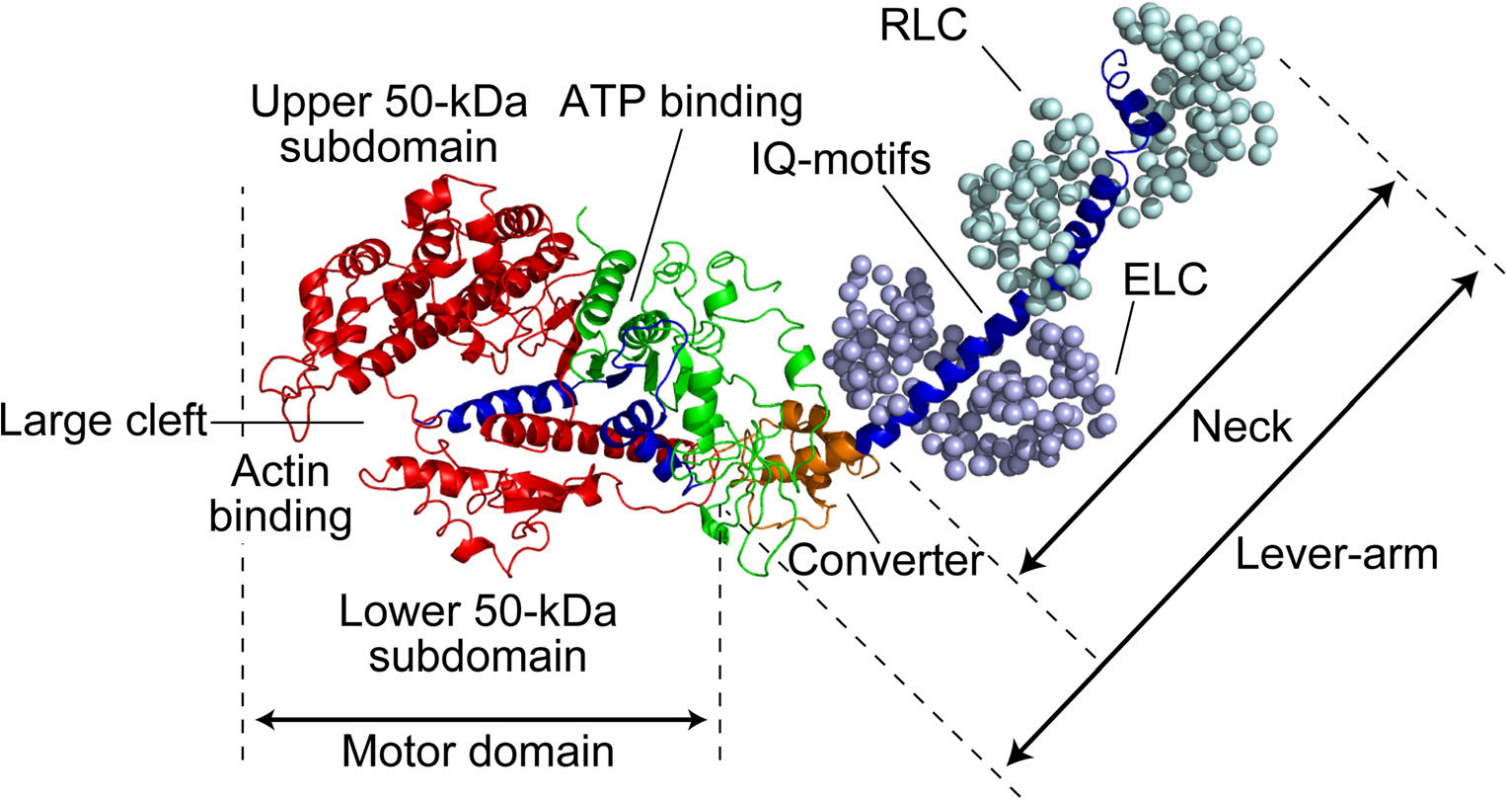
Crystal structure of skeletal myosin II S-1. The heavy chain of myosin is shown as a ribbon diagram, while two light chains [i.e. essential light chain (*ELC*) and regulatory light chain (*RLC*)] are shown as a sphere diagram. The 25-, 50-, and 20-kDa regions are colored *green*, *red*, and *blue*, respectively. The converter domain in the 20-kDa region is additionally colored with *orange*, after which IQ motifs follow

The myosin superfamily is largely categorized into the monomeric and dimeric types (Pecham 2011), although some myosins can adopt both forms depending on the environment (Park et al. 2006; Yu et al. 2009; Tokuo et al. 2007; Umeki et al. 2011) and both types of isoforms sometimes exist in the same class (e.g., monomeric yeast Myo4p and dimeric yeast Myo2p belonging to class V; Bookwalter et al. 2009). In stable dimeric myosins, the N-terminal head is followed by a coiled-coil tail domain for dimerization, while in monomeric myosins it is followed by a tail domain or a stable, single α-helical (SAH) domain (Knight et al. 2005) and then by a tail domain. The C-terminal tail end generally serves as a cargo-binding domain (Fig. 1). In this regard, dimeric myosin II is rather unique. It does not possess a cargo-binding domain and assembles into bipolar thick filaments mediated by a long coiled-coil tail. The tail and cargo-binding domains have diverse amino acid sequences, depending on the intracellular function of myosin (e.g., Sellers 2000), while the head region comprising a motor domain and a neck domain is generally common. The motor domain contains two functional sites, the actin-binding and nucleotide-binding sites which communicate with each other. The neck domain comprises a variable number of IQ motifs that bind calmodulin or myosin light chains. The neck is connected to the so-called converter region in the head, and these two as a single entity is called the lever-arm. When the SAH domain follows the neck, it is also included in the lever-arm because it functionally extends the length of the canonical lever-arm (Baboolal et al. 2009). The heavy chain in the head comprises three parts (25, 50, and 20–23 kDa) aligned in this order from the N-terminus that were revealed by limited tryptic cleavage of subfragment-1 (S-1) of skeletal myosin II (Mornet et al. 1979; Mornet et al. 1981). These three parts connected to two surface loops are arranged as shown in Fig. 2 in the three-dimensional (3D) structure of the head (Rayment et al. 1993a; Houdusse et al. 2000). This 3D arrangement of the three parts is largely common to all classes of myosin. The most prominent feature is the presence of a large cleft dividing the 50-K subdomain into lower and upper 50-K subdomains. The outer cleft regions of both lower and upper 50-K subdomains bind to actin mainly through loops. The ATP-binding site is located at the inner cleft region, around which all the three parts face each other. Interestingly, the 3D structure of the catalytic core is very similar to the entire motor domain structure of kinesin despite complete dissimilarity in the amino acid sequence, suggesting that they share a similar force-generating strategy (Kull et al. 1996).

Single-headed myosin S-1 binds to actin at a given angle relative to the actin filament. The bound single head (both motor and neck domains) often extends towards the plus end of actin at least in the nucleotide-free and ADP-bound conditions (Rayment et al. 1993b; Jontes et al. 1995). This orientation towards the plus end of actin is called “arrowhead orientation” (Huxley 1963). Among all members of the myosin superfamily, only myosin VI is known to move towards the minus (barbed) end of actin (Wells et al. 1999). The motor domain of a single-headed myosin VI construct also binds to actin in the arrowhead orientation. However, its neck domain is largely kinked relative to the motor domain, so that the neck domain orients slightly towards the minus end of actin (Wells et al. 1999). The swinging lever-arm hypothesis presumes that the lever-arm rotates so that a minute change in the motor domain is amplified to a large displacement of the lever-arm’s distal end. When this presumption is applied to myosin VI, its reversal movement must be achieved by the rotation of the lever-arm in the direction opposite to that of the other myosins (Wells et al. 1999). This is consistent with the kinked neck domain of myosin VI and also with the reversal movement of a myosin I construct with an artificial neck which is kinked towards the reverse-arrowhead orientation (Tsiavaliaris et al. 2004).

**Fig. 3:**
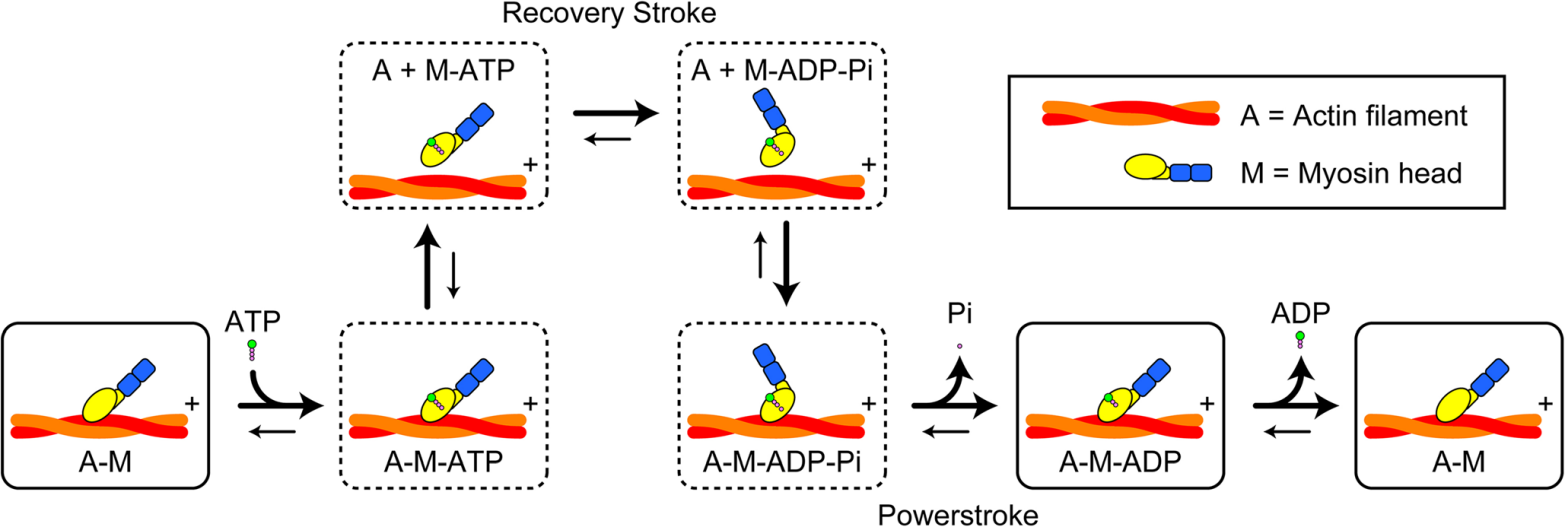
Scheme of muscle actomyosin ATPase reaction including structural model. + indicates the plus end of actin. *Boxes with solid lines and broken lines* represent the strong- and weak-affinity states, respectively. The powerstroke is considered to occur mainly in the transition from the A–M–ADP–Pi state to the A–M–ADP state. The recovery stroke is considered to occur in the transition from the M–ATP state to the M–ADP–Pi state

The actomyosin ATPase reaction proceeds as shown in Fig. 3. Immediately after binding to ATP, the actin-bound head detaches from actin, followed by quick hydrolysis of the bound ATP to ADP–Pi. The ADP–Pi-bound head has a low affinity for actin (yet a somewhat stronger affinity than the ATP-bound head). When the ADP–Pi-bound head is attached to actin, the bound Pi dissociates from the head, followed by the formation of a strongly-bound complex of A–M–ADP (A and M denote actin and myosin head, respectively), and then by ADP dissociation. The nucleotide-free head also has a strong affinity for actin. These complexes with strong affinities are called “rigor complexes”. Thus, a myosin head has two states with respect to its affinity for actin, the weak- and strong-binding states. The main role of actin in the ATPase reaction is to accelerate the otherwise very slow Pi and ADP dissociation from a myosin head. The degree of acceleration for Pi dissociation and ADP dissociation varies among the classes of myosin. The processivity occurs in dymeric myosins when the lifetime of A–M–ADP predominates over the other states (De La Cruz et al. 1999).

In the prevailing view on myosin motility, the chemo-mechanical coupling is considered to occur as follows (see Fig. 3). The myosin head undergoes conformational changes upon ATP binding and hydrolysis to ADP–Pi. Myosin then swings back the lever-arm (“recovery stroke”) (Geeves and Holmes 1999). The angle change between the motor and neck domains caused by this recovery stroke facilitates the binding of the head to actin in the reverse arrowhead orientation (note that the motor domain still binds to actin in the arrowhead orientation). The A–M–ADP–Pi state, often termed as the “pre-powerstroke state”, is considered to be a higher-energy state. Upon Pi release, tension for driving the lever-arm swing is generated, the lever-arm actually swings to execute a powerstroke, and then a lower-energy state of A–M–ADP (the post-powerstroke state) is formed. Thus, in this view, the chemical energy liberated by ATP hydrolysis is used mainly in the Pi-release step.

## Progress of motility assays

### The situation before the single-molecule era

Almost the entire enzymatic kinetics of actomyosin has been obtained from muscle myosin. In most of kinetics measurements, soluble forms of myosin, S-1 and heavy meromyosin (HMM), were used to simplify interpretations of the data. Then, the enzymatic kinetics and the structural characteristics of myosin were combined to understand how the myosin motor might work. Although the overall feature of the enzymatic kinetics was successfully revealed, no mechanical constraints were imposed to myosin molecules interacting with actin filaments in vitro. Therefore, it was difficult to deduce the most important characteristic of the mechano-enzyme, namely how does mechanical strain imposed on myosin affect the ATPase reaction? Moreover, the whole cycle of the ATPase reaction could not be tracked down in one measurement because the initial synchrony among molecules becomes obscured by the stochastic nature of the reaction. Therefore, many kinetic constants derived for the entire reaction scheme were obtained in separate experiments where the reactions were started with different initial states. In some cases, it was difficult to prepare adequate initial states. As such, a quantitative reaction scheme was obtained by “pasting” many results together that were not necessarily obtained under similar solution conditions.

The mechanical behavior of myosin interacting with actin filaments could not be observed in solution experiments. To acquire this information, muscle fibers and myofibrils were subjected to X-ray diffraction (Huxley et al. 1981, 1983), fluorescence polarization (Nihei et al. 1974; Borejdo and Putnam 1977), electron paramagnetic resonance (EPR) spectroscopy (Thomas and Cooke 1980), and force analyses (Ford et al. 1977). At a very early stage intrinsic tryptophan fluorescence or fluorescent ATP analogues were used for fluorescence polarization studies (dos Remedios et al. 1972a, b). Later, fluorescence and spin polarization probes were introduced to a specific cysteine, SH1, in the motor domain. This was possible because SH1 is most reactive of all myosin cysteines (Sekine and Kielly 1964) and indeed of all proteins in skinned muscle fibers (Borejdo and Putnam 1977), and therefore could be selectively labeled. Unfortunately, it later became known that the SH1-labeled myosin does not function as a motor (Root and Reisler 1992; Marriott and Heidecker 1996; Bobkov et al. 1997). Extensive muscle fiber X-ray diffraction studies were performed but no clear evidence for cross-bridge movement was obtained. In the light of numerous spectroscopic and structural studies, the massive part of an actin-bound myosin head proximal to actin was suggested to remain unchanged during the powerstroke, whereas the distal neck region with smaller mass than the proximal one was suggested to change its orientation (Cooke 1986). In this case it would be difficult to distinctly detect this change in the fiber diffraction patterns. Moreover, the time in which a myosin head interacts with an actin is very short in the ATPase cycle, making it difficult to record the dynamics of cross-bridges.

The molecular structures of myosin heads in different nucleotide conditions were successfully revealed at submolecular level using electron microscopy (e.g., Walker and Trinick 1988; Katayama 1989; Pollard et al. 1993; Takács et al. 2010) and at the atomic level using X-ray crystallography (e.g., Rayment et al. 1993a; Fisher et al. 1995; Dominguez et al. 1998; Houdusse et al. 2000). However, as a matter of course, these structures were static snapshots. Moreover, even with the current state of technology, it is still difficult to obtain the atomic structure of actin-bound myosin head, particularly the structure in the power-stroking state (Rayment et al. 1993b; Lorenz and Holmes 2010).

### In vitro motility assays

**Fig. 4:**
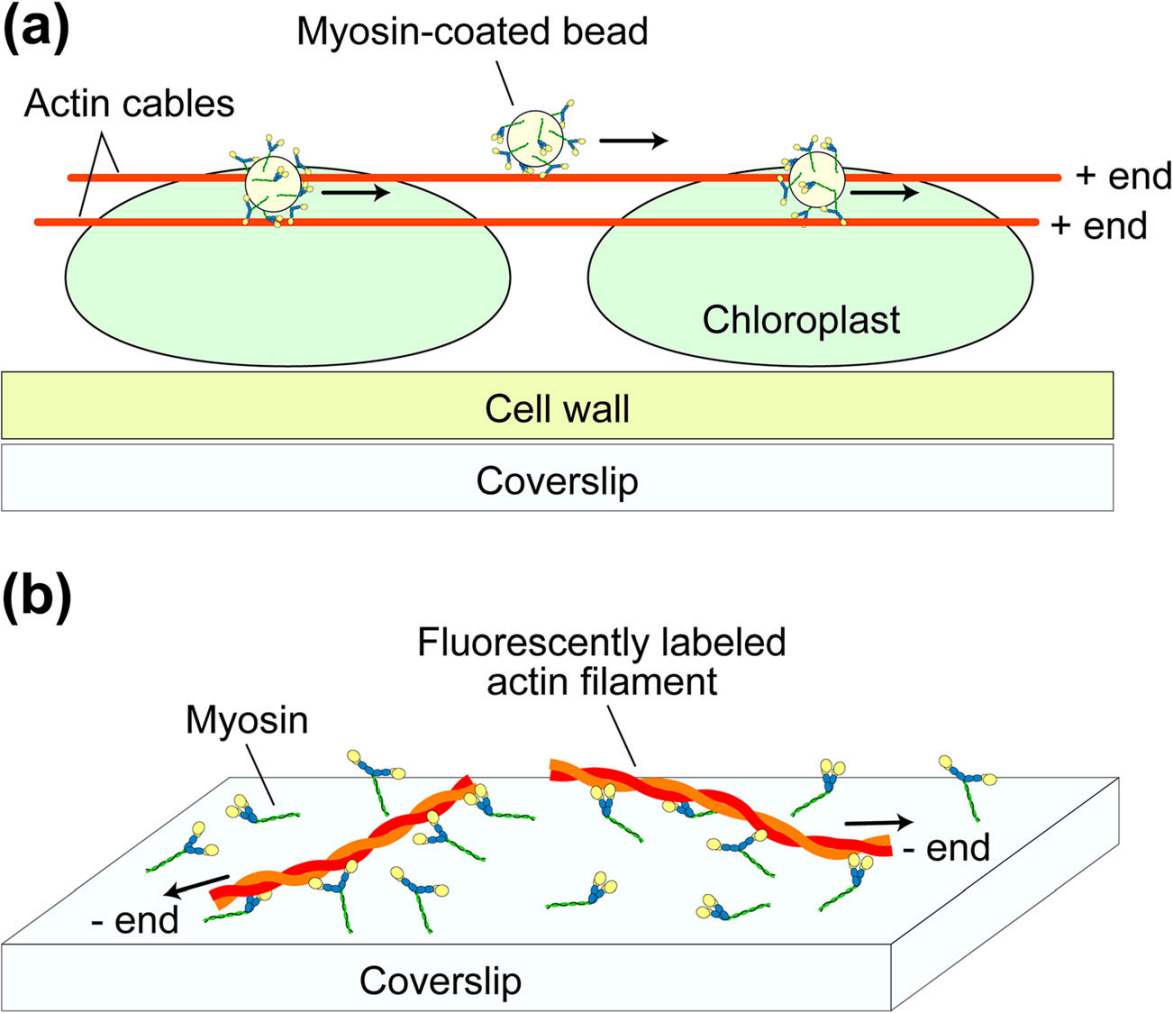
Schematics showing in vitro motility assay systems for actomyosin. **a** Myosin-coated bead assay. The myosin-coated fluorescent beads are subjected to the polar arrays of actin cables naturally formed on chloroplastd of the alga *Nitella*, and movement of the beads are observed under a florescent microscope. **b** Actin filament gliding assay. Myosin molecules are attached to the surface of a nitrocellulose-coated coverslip and gliding motion of the fluorescently labeled actin filaments are observed under a fluorescence microscope

To overcome some of the above adversities, experimental techniques called in vitro motility assays were developed in the early 1980s, as advanced versions of previous methods developed in the 1970s (Oplatka and Tirosh 1973; Kuroda and Kamiya 1975). Dark-field microscopy was performed that detected the movement of fibrils that were squeezed out of the giant alga *Nitella* cell, washed with buffer, and mixed with ATP (Higashi-Fujime 1980). Next, fluorescence microscopy was conducted using a more purified system where skeletal myosin-coated fluorescent beads were observed to slide on a substratum of polar arrays of actin cables derived from giant alga, *Nitella* (Fig. 4a) (Sheetz and Spudich 1983). The movement was ATP-dependent and blocked by inactivation of the myosin heads using N-ethylmaleimide. This study revealed that the bipolar thick filament structure is not necessary for generating force and movement. Remarkably, this assay system was much simpler and more robust than previous in vitro motility assay systems (Oplatka and Tirosh 1973; Kuroda and Kamiya 1975; Higashi-Fujime 1980; Yano et al. 1982), and this facilitated quantitative analyses of the myosin motility. Using fluorescently labeled single actin filaments, their thermal bending motion was observed to increase in the presence of HMM and ATP (Yanagida et al. 1984). Also was demonstrated that the sliding movement of individual actin filaments on myosin-coated surfaces occurred at velocities similar to those recorded in muscles (Fig. 4b) (Spudich et al. 1985). Moreover, actin filaments were observed to slide on one-headed myosin filaments, indicating that a two-headed structure is not needed for motility (Harada et al. 1987). This result was reinforced by the observation of actin filaments gliding over myosin S1-coated surfaces (Toyoshima et al. 1987). Thus, motor activity inherent in the head was established, which ruled out some models of muscle contraction.

#### In vitro motility studies on chemo-mechanical relationship

Understanding how actomyosin ATPase kinetics determines the mechanical performance of this motor system (magnitude of generated force and sliding velocity) became a central focus of muscle research. Even in the in vitro motility assays, single actin filament sliding and the translational motion of myosin-coated beads are propelled by multiple myosin heads. Therefore, the chemo-mechanical relationship was not straightforward. Nonetheless, the in vitro motility assays made the analysis more precise and more extensive than previous assays where muscle fibers were studied. The in vitro motility assays allowed us to investigate actomyosin motility under wide varieties of solution conditions with myosins and actins from diverse species and cell types. The velocity of actin filament sliding over myosin-coated surfaces and the velocity of myosin-coated beads along actin filaments were shown to be analogous to the speed of unloaded shortening of muscle fibers (Sheetz et al. 1984; Spudich et al. 1985; Kron and Spudich 1986; Homsher et al. 1992). Despite the reported analogy, many studies using different types of myosin II found no direct correlation between the steady-state actomyosin ATPase activity and the rate of movement measured by the in vitro motility assays (Umemoto et al. 1989; Lowey et al. 1993; Uyeda et al. 1994; Vale et al. 1984; Higashi-Fujime 1991). Nor was direct correlation observed using different nucleotide triphosphates (NTPs) condition (Shimizu et al. 1991; Higashi-Fujime and Hozumi 1996). In these studies, one concentration of actin and one concentration of NTPs were used in the measurements of the enzymatic activities. In similar studies where the actin concentration was varied and a fixed concentration of NTPs was used, a moderate correlation was shown between the motor activity and the substrate turnover rate (Pate et al. 1993a; Regnier et al. 1998).

**Fig. 5:**
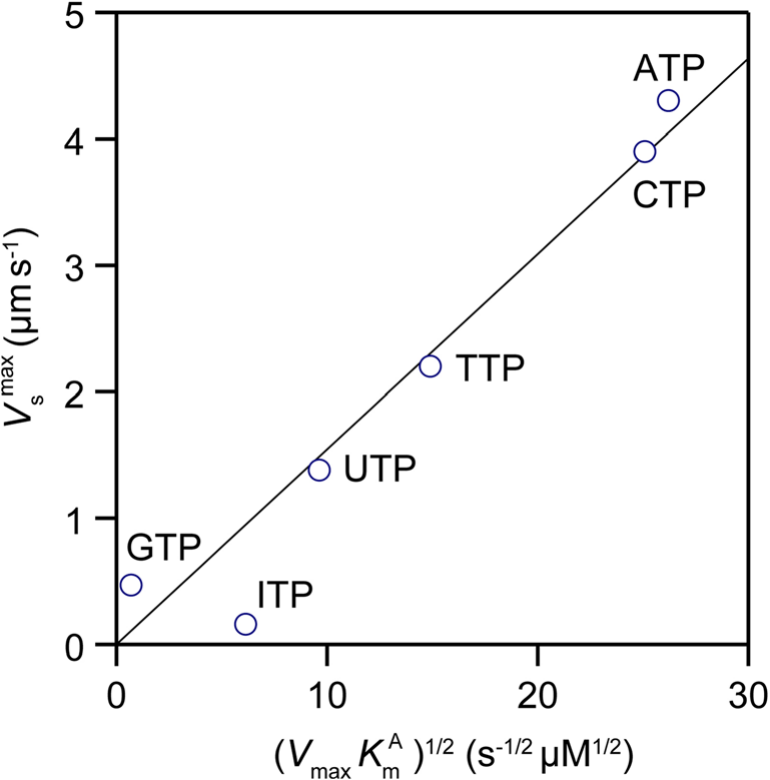
Relationships between the maximum sliding velocity (*V*_s_^max^) and the actomyosin NTPase kinetics parameters, *K*_m_ for actin (i.e. *K*_m_^A^) and maximum NTPase rate *V*_max_. The nucleotide substrates used are indicated by the side of the corresponding data plots

A more extensive study was conducted for rabbit skeletal acto-HMM using six naturally occurring NTPs with Mg^2+^, different divalent cations with ATP, and Mg^2+^-ATP at various ionic strengths (Amitani et al. 2001). The maximum substrate turnover rates (*V*_max_), *K*_m_ for actin (*K*_m_^A^), and *K*_m_ for metal^2+^-NTPs (*K*_m_^N^) were obtained while the velocity of actin filament gliding on HMM-coated surfaces was measured under the same buffer and substrate conditions used for the measurements of substrate turnover rate. A large dataset acquired revealed a direct linear relationship, except when different divalent cations were used with ATP. The maximum sliding velocity (*V*_s_^max^) extrapolated to the infinite concentration of substrates was directly proportional to (*V*_max_ *K*_m_^A^)^1/2^ (Fig. 5). This experimental correlation was analyzed based on a force–balance model, where the average sliding force (active force) produced by a productive head, <*f*_s_>, is balanced with the average resistive force produced by an actin-bound non-productive head, <*f*_r_>, which is directly proportional to the gliding velocity of the actin filament. At infinite substrate concentration, <*f*_s_> equals *pT*_p_/*T*_c_, where *p* is the magnitude of powerstroke (force spike), *T*_p_ is the duration of single powerstroke, and *T*_c_ is the substrate turnover time. The power stroke speed (*d*/*T*_p_; where *d* is actin filament displacement by each powerstroke) should equal the maximum sliding velocity (*V*_s_^max^) (Uyeda et al. 1991). Thus, the following relationship is derived:

1 $$ <{f}_{\mathrm{s}}>= pd{V}_{\max }/{V}_{\mathrm{s}}^{\max }. $$

At infinite substrate concentration, the resistive force averaged over the substrate turnover time <*f*_r_> is expressed as:

2 $$ <{f}_{\mathrm{r}}>=\frac{1}{2}\lambda \varGamma {V}_{\mathrm{s}}^{\max }/{k}_{\hbox{--} }, $$

where λ is the fraction of the actin-bound non-productive state over all states, *Γ* is an elastic constant of the resistive head, and *k*_–_ is the dissociation rate of the resistive head. Supposing that the resistive state is the weakly bound state, *k*_–_ approximately equals *k*_+_ *K*_m_^A^, where *k*_+_ represents the rate constant in which a detached head in the weak-binding state binds to actin. Thus, from Eqs. (1) to (2), the following relationship is derived:

3 $$ {V}_{\mathrm{s}}^{\max }={\left(2{k}_{+} pd\;{K}_{\mathrm{m}}^{\mathrm{A}}{V}_{\max }/\lambda \varGamma \right)}^{1/2}. $$

As the fraction of the resistive state λ is likely to be proportional to *k*_+_, *V*_s_^max^ becomes directly proportional to (*V*_max_ *K*_m_^A^)^1/2^, which agrees well with the experimentally observed relationship (Fig. 5). The lifetime of the weakly bound state is very short but this state occurs many times within a substrate turnover cycle. Thus, the weakly bound state becomes a predominant resistive state. This force balance model could also be extended to the case where actin filaments moved at various substrate concentrations by including the resistive force produced by rigor heads; thus, it could derive the dependence of sliding velocity on the substrate concentration. Its derivation showed that the dependence of the sliding velocity on the substrate concentration [S] followed a modified Michaelis–Menten equation of *V*_s_ = *V*_s_^max^/{1 + (*K*_m_^N^/[S])^n^} (here, 1 < n < 2), which again agreed well with the experimentally obtained dependence under various conditions (Amitani et al. 2001).

#### Step size

The next fundamental question to tackle experimentally was the translocation distance that is propelled by actin—myosin interaction during an ATPase cycle (i.e. step size). The swinging lever-arm hypothesis predicts the size to be ∼10 nm. However, as mentioned above, the in vitro actin gliding or myosin-bead gliding assay is not a single-molecule assay. Moreover, the duration of the working stroke that occurs in an ATPase cycle has to be measured to estimate the step size but cannot be measured directly. Consequently, various values were reported for the step size: ∼8 nm (Toyoshima et al. 1990), ∼10 nm (Pate et al. 1993b), 10–28 nm (Uyeda et al. 1991), and >60 nm (Yanagida et al. 1985; Harada et al. 1990). To resolve this large discrepancy, the step size had to be directly measured at nanometer and millisecond spatiotemporal resolution. This requirement gave birth to the so-called “single-molecule measurement assays” capable of observing and manipulating single biological molecules in action.

### Single-molecule measurements

**Fig. 6:**
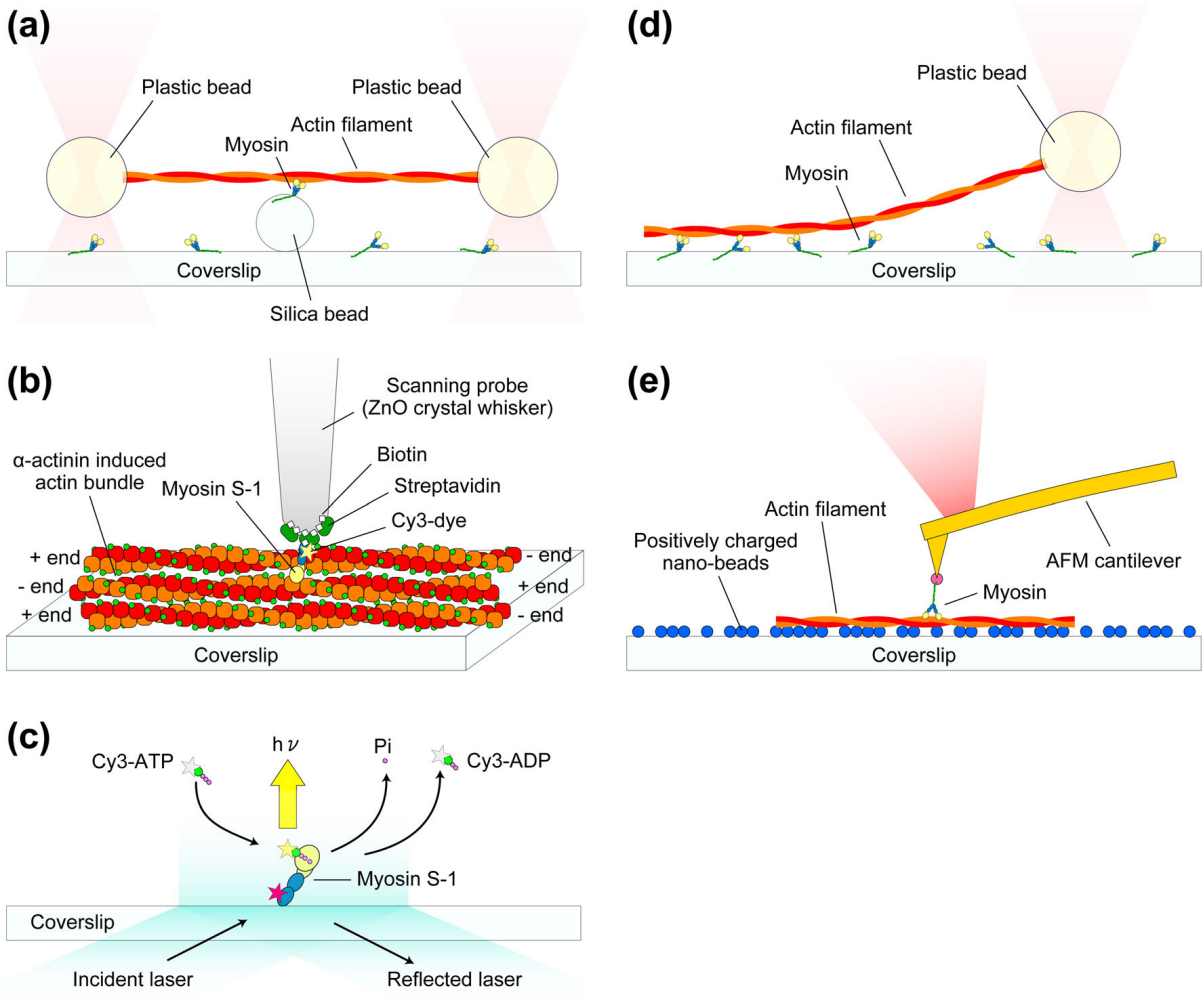
Schematics showing typical single-molecule measurement systems for studying myosin–actin interactions. **a** Dual-beam laser trap assay. Each end of an actin filament is attached to a plastic bead, and the beads are trapped by the focused laser beams. The trapped actin filament is brought to a single myosin molecule attached to a silica bead. **b** Mechanical measurement system with the use of a thin glass micro-needle as a force sensor. A ZnO crystal whisker is attached to the end of a glass micro-needle (not shown in this schematic). Fluorescently labeled single myosin S-1 is attached to the apex of the ZnO crystal whisker via a specific binding of biotin-streptavidin. The tip is brought onto actin bundles formed by α-actinin. The displacements of the S-1 resulting from the interaction with actin are monitored by the detection of glass micro-needle deflection. **c** TIRFM experiment for visualizing individual ATP turnovers by single myosin S-1. The light chain of S-1 molecule is labeled with Cy5 (indicated by the *red star*) for the confirmation of whether or not the observed ATP turnovers are those by a single S-1 molecule. Once Cy3-ATP binds to the S-1, the large Brownian motion of the Cy3-ATP stops and therefore its fluorescent spot becomes visible. **d** Single beam assay for the measurement of unbinding force of a single myosin–actin bond. **e** AFM measurement of rupture force and rupture distance of a single actin–myosin bond

Optical tweezers are typically used in single molecule measurements. A micrometer-sized dielectric particle such as a polystyrene bead is trapped by a focused laser beam (Ashkin and Dziedzic 1985; Neuman and Block 2004) and a trapped particle can be freely moved by changing the focal position of the laser beam. The position of the trapped particle in the XY plane perpendicular to the laser beam can be measured with ±<1 nm precision. For the trapped particle, the focused laser beam works as a 3D spring with a spring constant of 0.01–10 pN/nm. When the particle is displaced by an external force in the XY plane, the magnitude of the external force applied can therefore be measured at high precision. This optical trap nanometry is now routinely used to study biological molecules (Svoboda and Block 1994; Mehta et al. 1998; Wang 1999; Bustamante et al. 2000; Kuo 2001). Step size and the force produced by a single myosin head were directly measured using a dual-beam laser trap (Finer et al. 1994; Molloy et al. 1995; Guilford et al. 1997; Tanaka et al. 1998; Veigel et al. 1999). Each end of an actin filament was attached to a particle. The two particles are caught in the dual-beam laser trap, and then the actin filament is allowed to interact with a single myosin molecule immobilized on a surface (Fig. 6a). The measured step size ranged between 3.5 and 15 nm but the extent of discrepancy between laboratories was much smaller than previous estimates made using actin gliding assays. It was pointed out by Yanagida’s group that the measured step size tended to be affected by the interaction angle between myosin and actin. With careful design, the measured step size was shown to be dependent on the interaction angle (Tanaka et al. 1998). When an actin filament was aligned parallel with a myosin filament with a low myosin head density, a relatively large step size of 10–15 nm was obtained (Tanaka et al. 1998).

A very fine glass micro-needle was also used by Yanagida’s group as a force transducer for single molecule mechanical measurements. A single actin filament was attached to the free end of a micro-needle and brought into contact with an immobilized single myosin molecule. The displacement of the micro-needle caused by the myosin–actin interaction was measured (Kishino and Yanagida 1988; Ishijima et al. 1991; Ishijima et al. 1996). Using a low needle stiffness (0.09 pN nm^−1^) and near zero load, the average of single displacement spikes was ∼20 nm (Ishijima et al. 1996). However, the spatiotemporal resolution was not high enough to precisely detect the displacement spikes. Then, an experimental system was further developed (Fig. 6b); a ZnO crystal whisker with a tip radius of ∼15 nm was attached to a fine glass needle mounted on a three-dimensional piezoelectric actuator. A single myosin S-1 fluorescently labeled at the regulatory light chain was attached to the end of the ZnO whisker. The molecule was brought into contact with actin bundles formed by chicken gizzard α-actinin, where the actin filaments were aligned in anti-parallel (Meyer and Aebi 1990). Using a glass needle with very small stiffness (0.01–0.03 pN nm^−1^), the spatial resolution of 2.0 nm and the temporal resolution of <0.2 ms were obtained (Kitamura et al. 1999, 2005). Using this technique, an average step size of ∼13 nm was measured, but surprisingly several (2–5) 5.5-nm successive substeps were also detected within each step. This size, 5.5 nm, coincides with the distance between adjacent actin monomers in a filament. More surprisingly, the dwell between substeps was independent of the ATP concentration (0.1 and 1 μM). Therefore, it was concluded that the multiple substeps produced by a single myosin head occurred during just one cycle of ATP hydrolysis. This conclusion was reinforced by theoretical studies (Terada et al. 2002; Takano et al. 2010). However, the experimental result has never been reported by other groups.

Total internal reflection fluorescence microscopy (TIRFM) has also been used for single-molecule measurements (Park et al. 2007; Murcia et al. 2007; Joo et al. 2008). When an incident laser light is totally reflected at the glass–water interface, an evanescent field is generated that decays exponentially from the interface, penetrating to a depth of ∼100 nm into the solution. Since only fluorophores located in the evanescent field are excited, the background light level is very low, allowing the visualization of single fluorophores (Fig. 6c). Using a Cy3-labeled ATP, the dynamic events of its binding to and dissociation from single myosin S-1 molecules were visualized (Funatsu et al. 1995). The dissociation rate estimated from the lifetime of the bound Cy3-nucleotide agreed with the biochemically measured rate of Cy3-ATP turnover by S-1. This success opened up a new opportunity to simultaneously measure the chemical and mechanical events in single myosin molecules. Using one-headed skeletal muscle myosin assembled with myosin rod and a setup where TIRFM and a dual-beam laser trap were combined, mechanical events of actin–myosin dissociation/force generation (displacement) and chemical events of binding/dissociation of Cy3-ATP (or Cy3-ADP) were simultaneously observed (Ishijima et al. 1998). The timing of Cy3-ATP binding well coincided with the timing of myosin head dissociation from actin. However, in more than 50 % of 85 detected events, the actin filament was displaced 0.3–1.8 s after the dissociation of Cy3-nucleotide from the myosin head. This surprising result suggested that myosin has a hysteresis or memory state in which the chemical energy liberated by ATP hydrolysis is stored even after ADP dissociation. However, photobleaching of bound Cy3-nucleotide also looks like it is dissociated from the myosin head. Therefore, their interpretation could not be statistically verified. The step size measured in this study (∼15 nm) was consistent with their previously reported values.

Single-molecule measurements quantified other mechanical properties of actin, myosin, and their interactions. The tensile and torsional rigidity of a single actin filament was determined using a glass micro-needle technique (Kishino and Yanagida 1988) and optical tweezers (Tsuda et al. 1996), respectively. The unbinding force between an actin filament and a single myosin head in the nucleotide-free condition was ∼9 pN using an optical tweezers method where an actin filament was pulled approximately along its length (Fig. 6d) (Nishizaka et al. 1995). It was also measured to be ∼15 pN with AFM, where a single myosin head was pulled in the direction perpendicular to the actin filament (Fig. 6e) (Nakajima et al. 1997).

As described above, no complete consensus was reached among research groups regarding the myosin step size taken for each ATP hydrolysis, and hence also as to the swinging lever hypothesis. The discrepancy was due partly to a relatively small step size occurring in a short time that was technically difficult to measure.

## Myosin V

The technical difficulty mentioned above was significantly removed by the discovery of M5 that possesses unique structural and biochemical features. M5 is involved in organelle and mRNA transports as well as in membrane trafficking. Its cellular functions and regulations are well described elsewhere (Reck-Peterson et al. 2000; Taylor 2007; Sellers and Knight 2007; Sellers and Weisman 2008), so here we focus on its motor mechanism.

### Structural and biochemical features of M5

The most prominent structural feature of two-headed M5 is its neck domain (∼24 nm) (Cheney et al. 1993) which is approximately three-fold longer than that of myosin II (∼8 nm). If M5 really follows the swinging lever-arm hypothesis, a step size markedly larger than that of muscle myosin II should be observed. M5 was biochemically shown to dominate in a high actin-affinity state (ADP-bound state) during its ATPase cycle (De La Cruz et al. 1999), in stark contrast with muscle myosin II where a weak affinity sate (ADP–Pi-bound state) dominates.

### Processive movement

A dual-beam optical trap was used to show that a suspended actin filament successively moved 3–6 times when it interacted with a single M5 molecule attached onto a bead (Mehta et al. 1999). The dwell time depended on the ATP concentration, and therefore, successive step movements differed from the sub-stepping observed previously with myosin II (Kitamura et al. 1999, 2005). This was the first evidence that M5 is a processive motor. Importantly, the step size was ∼36 nm, consistent with the swinging lever-arm hypothesis. A much longer processive run (over several microns) of single M5 molecules along surface-immobilized actin filaments was demonstrated with TIRFM (Sakamoto et al. 2000). In a single-beam optical trap, a trapped bead coated with M5 at low density moved processively with ∼36-nm steps along a surface-immobilized actin filament (Rief et al. 2000). The ∼36-nm step size corresponds to the head-head distance shown in electron micrographs of M5 with two heads bound to the same actin filament (Walker et al. 2000). From dwell time analyses of the data, the stepping kinetics was shown to be limited by ADP release. In this study, a hand-over-hand model was first proposed, i.e. the two heads alternately switch the leading and trailing positions in every ATPase cycle.

The processivity is consistent with the cellular functions of M5 (e.g., organelle transports). The motility of kinesin, a cargo-carrying microtubule-based processive motor, has been well characterized although its step size is relatively small (∼8 nm) (Vale et al. 1985; Block 2007; Hirokawa et al. 2009). M5 possesses both processivity and a large step size, which were of great advantage in studying details of how M5 moves.

### Inhibitory state of M5

In one of the above studies on the full length of M5 (Sakamoto et al. 2000), the ATP turnover rate was measured biochemically under the same buffer solution conditions (without Ca^2+^) used for the observation of long processive movement. From the maximum actin-activated ATPase rate measured (∼1.2 s^−1^/head) and the maximum velocity of M5 movement observed with TRIFM (1 μm s^−1^), M5 appeared to travel ∼400 nm per ATPase cycle. This long travel distance implied that a large fraction of M5 (more than 90 %) would be in a catalytically inhibited or low-activity state. Any M5 not in this state would have a much higher ATPase activity (14 s^−1^/head). Interestingly, in previous studies an ATPase rate as high as 14 s^−1^/head was reported for single-headed M5 constructs in the absence of Ca^2+^ (De La Cruz et al. 1999; Trybus et al. 1999) as well as for the full length of M5 in the presence of μM Ca^2+^ (Nascimento et al. 1996). Later, this mystery of the fast movement (1 μm s^−1^) with a low ATPase activity (∼1.2 s^−1^/head) was solved by a finding that M5 often takes a folded conformational state in the absence of Ca^2+^, in which the C-terminal cargo-binding domains bind to the heads, resulting in the inhibition of the ATPase and motor activities (Wang et al. 2004; Krementsov et al. 2004; Li et al. 2006; Liu et al. 2006; Thirumurugan et al. 2006; Olivares et al. 2006). Also, several other myosins such as myosin VII and X are now known to be regulated by head–tail interactions (Yang et al. 2009; Umeki et al. 2009, 2011). Intramolecular folding appears to be a common mechanism to inhibit the activity of motor proteins as a similar conformational transition was shown previously with smooth muscle myosin (Trybus et al. 1982) and kinesin (Hackney et al. 1992). However, a recent study of smooth muscle myosin suggests that an intramolecular interaction between the heads would be a key event in the regulation, rather than a head–tail interaction (Baumann et al. 2012).

### Processive hand-over-hand movement

The ∼36-nm step size of M5 corresponds to the long pitch pseudo-repeat of the actin helix, suggesting that M5 moves on a plane along an actin filament. This inference was certainly proven to be the case by the observation of the movement of an M5-attached bead on a suspended actin filament. The bead advanced in a left-handed spiral trajectory with one revolution per 2.3 μm, i.e. only 5.6° rotation per step (Ali et al. 2002).

The next study to be done was to confirm the tight relationship between the neck length and the step size, using M5 constructs with different neck lengths. It was actually confirmed that the step size is directly proportional to the neck length (Purcell et al. 2002; Sakamoto et al. 2003; Moore et al. 2004; Sakamoto et al. 2005). Thus, the swinging lever-arm hypothesis was nearly secured.

Next, many studies focused on the experimental verification and close inspection of the hand-over-hand movement previously proposed (Rief et al. 2000). To this end, various light microscopy techniques or fluorescent probes were introduced to TIRFM: fluorescence imaging with one nanometer accuracy (FIONA) (Yildiz et al. 2003), fluorescence polarization (Forkey et al. 2003), two-color imaging with Q-dots (Warshaw et al. 2005), single-molecule high-resolution colocalization of fluorescent dyes (Churchman et al. 2005), a combination of FIONA and fluorescence polarization (Syed et al. 2006), and defocused orientation and position imaging (Toprak et al. 2006). All these studies revealed that in fact M5 walks in a hand-over-hand manner. Not only translational position changes of the two heads but also angular changes of the lever-arm relative to actin were observed where only one of the two lever-arms was labeled with a fluorescent probe. The value of the angle change (∼60°) was also consistent with a hand-over-hand movement (Forkey et al. 2003; Syed et al. 2006; Toprak et al. 2006).

These studies supported the hand-over-hand model but only the dwell states were observed when both heads were bound to actin. Therefore, little was known about the fleeting intermediate between the dwell states because the time resolution was low (∼33 ms or longer). To increase the time resolution to 1 ms or less, the following imaging methods were employed: dark field imaging of a 40-nm gold nanoparticle attached to one of the lever-arms (Dunn and Spudich 2007) and recording of light-scattering from a 200-nm M5-coated latex bead traveling in an interference fringe pattern (Cappello et al. 2007). Alternatively, the action of walking M5 was slowed down by attaching a large fluorescent microtubule to one of the lever-arms and observed by conventional fluorescence microscopy (Shiroguchi and Kinosita 2007). All three studies revealed that the detached head underwent extensive rotational Brownian motion before landing on a site ahead of the leading head, suggesting that the detached head is unlikely to interact with actin during the Brownian motion. Moreover, it was suggested that the Brownian motion is likely to be biased forward by the orientational change (swing) of the actin-bound leading head, by which the detached head eventually finds an actin ahead of the leading head to become a new leading head. Thus, a clearer picture of the processive hand-over-hand movement was obtained.

### Asymmetry of enzymatic kinetics between two heads

The processive hand-over-hand movement suggests that only the trailing head can detach from actin. If so, there are two possibilities: (Case 1) ADP dissociation followed by ATP binding predominantly occurs at the trailing head, so that the ADP dissociation would be accelerated at the trailing head or retarded at the leading head; (Case 2) the ATP-binding rate would be enhanced at the trailing head or suppressed at the leading head. Between these possibilities, Case 1 appeared more likely as the ATPase rate is limited at the ADP release step. In either case, the kinetics asymmetry between the two heads would be engendered by head-to-head communications through intramolecular strain. This kinetics asymmetry was addressed by solution kinetics studies and optical-trap nanometry studies. In the solution kinetics studies various kinetics constants were compared for S-1 and HMM constructs of M5, although the two heads of M5-HMM do not necessarily interact with the same actin filament in solution (Rosenfeld and Sweeney 2004; Forgacs et al. 2008). These studies suggested retardation of ADP release from the leading head by ∼45-fold (Rosenfeld and Sweeney 2004) or up to 250-fold (Forgacs et al. 2008). The latter study used new fluorescent ADP and ATP analogues (deac-aminoADP and deac-aminoATP) (Webb et al. 2004) whose emission intensity increases by 20-fold upon binding to the active site of M5 (Forgacs et al. 2006). In the dual-beam trap experiments, the actin filament that was interacting with a surface-attached single-headed M5 molecule was pulled forward or backward to apply a force to the actin-bound head (Purcell et al. 2005; Veigel et al. 2002, 2005). The forward and backward forces were hypothesized to simulate forces experienced by the trailing or the leading head, respectively. Different designs of optical trap nanometry were also used to study the coordination between actin-bound two heads (Uemura et al. 2004; Clemen et al. 2005; Gebhardt et al. 2006; Oguchi et al. 2008). Most of these studies suggested that the intramolecular strain would coordinate two heads by decelerating ADP release from the leading head, whereas some studies suggested accelerated ADP release at the trailing head (Veigel et al. 2002, 2005), as proposed by a study of processive run of M5 perturbed by varying the nucleotide content (Baker et al. 2004).

In two-headed bound M5, the neck of the trailing head takes the arrowhead orientation, whereas the neck of the leading head takes the reverse arrowhead orientation. The leading head conformation must be mechanically distorted because single-headed M5 binds to an actin filament in an arrowhead orientation. In fact, electron micrographs of two-headed bound M5 showed that the leading neck was curved slightly outwards whereas the trailing neck was straight (Walker et al. 2000; Burgess et al. 2002; Oke et al. 2010). This conformational distortion seems consistent with the suggestion that the coordination of M5 stepping is performed by strain-mediated inhibition of ADP release from the leading head.

Finally, using two-color TIRFM, Sakamoto et al. (2008) simultaneously observed the stepping motion of fluorescently labeled M5 and the binding and dissociation of the fluorescent nucleotide (deac-aminoADP or deac-aminoATP). This observation directly revealed the preferential ADP dissociation from the trailing head, which was followed by a ∼36-nm step triggered by ATP binding. It also revealed that every M5 molecule always keeps at least one nucleotide (ADP in the leading head) during its stepping, even at low ATP concentrations.

### Discrepancies and unsolved questions in myosin V motility

The above description suggests there is now a consensus on how M5 steps. In every ATP hydrolysis cycle, the leading head swings to propel M5 forwards by ∼36 nm (Sakamoto et al. 2008). This large stride is realized by the long lever-arms of M5 (Purcell et al. 2002; Sakamoto et al. 2003; Moore et al. 2004; Sakamoto et al. 2005). The two heads alternately switch between the leading and trailing roles so that M5 walks hand-over-hand (Yildiz et al. 2003; Forkey et al. 2003; Warshaw et al. 2005; Churchman et al. 2005; Syed et al. 2006; Toprak et al. 2006). This style of movement is made possible by the intramolecular strain-mediated retardation of ADP release from the leading head (Rosenfeld and Sweeney 2004; Purcell et al. 2005; Forgacs et al. 2008; Oguchi et al. 2008; Sakamoto et al. 2008).

Nonetheless, several observations that are difficult to reconcile with this generally agreed view have been reported, mostly from the Yanagida lab. A recombinant two-headed M5 with a single IQ motif on each neck (∼4 nm long) was reported to move processively with ∼36-nm steps (Tanaka et al. 2002). It was claimed that the long-neck domain was not essential for both large step size and processivity of M5 but the motor domain alone determined the processivity and the large step size. It was also reported that single-headed M5 constructs underwent multiple successive large (∼32-nm) steps on an actin filament suspended by a dual-beam optical trap (Watanabe et al. 2004). They also underwent directional diffusion on surface-immobilized actin bundles with ∼5.5-nm substeps to develop an average displacement of ∼20 nm, which was independent of the neck length (2IQ and 6IQ motifs) (Okada et al. 2007), as previously observed with single-headed myosin II (Kitamura et al. 1999). Likewise, myosin VI with its short neck similar to myosin II, was reported to move with ∼36-nm steps (Rock et al. 2001; Nishikawa et al. 2002). However, among these reported irreconcilable conclusions, only the large step size of myosin VI was resolved. The SAHs were found in the proximal tail domain of myosin VI, which contribute to the lever-arm length (Baboolal et al. 2009; Spudich and Sivaramakrishnan 2010; Sweeney and Houdusse 2010b). The other discrepant results are still incomprehensible (Cyranoski 2000; Geeves 2002).

Even if the above general view of how M5 steps is completely right, there remain fundamental questions to solve. How is the chemical energy liberated by ATP hydrolysis used to generate the tension for forward movement? How are the tension generation and lever-arm swing coupled to the ATPase cycle? If they are coupled, which chemical transitions are involved in these mechanical events? These fundamental questions have long been explored in muscle contraction studies and some are considered to be resolved. Are there any inconsistencies when this consensus view is applied to the motility of M5? Numerous single-molecule measurements of M5 stepping have not answered these important questions.

The discrepancies mentioned above, as well as the remaining unsolved fundamental questions, have certainly proven that the level of “directness of measurement” is not yet high enough even with single-molecule optical measurements. Proteins molecules themselves are indeed invisible in these measurements, which can never be overcome even with diffraction-breaking super-resolution fluorescence microscopy (Egner and Hell 2005; Huang et al. 2009).

## HS-AFM

**Fig. 7:**
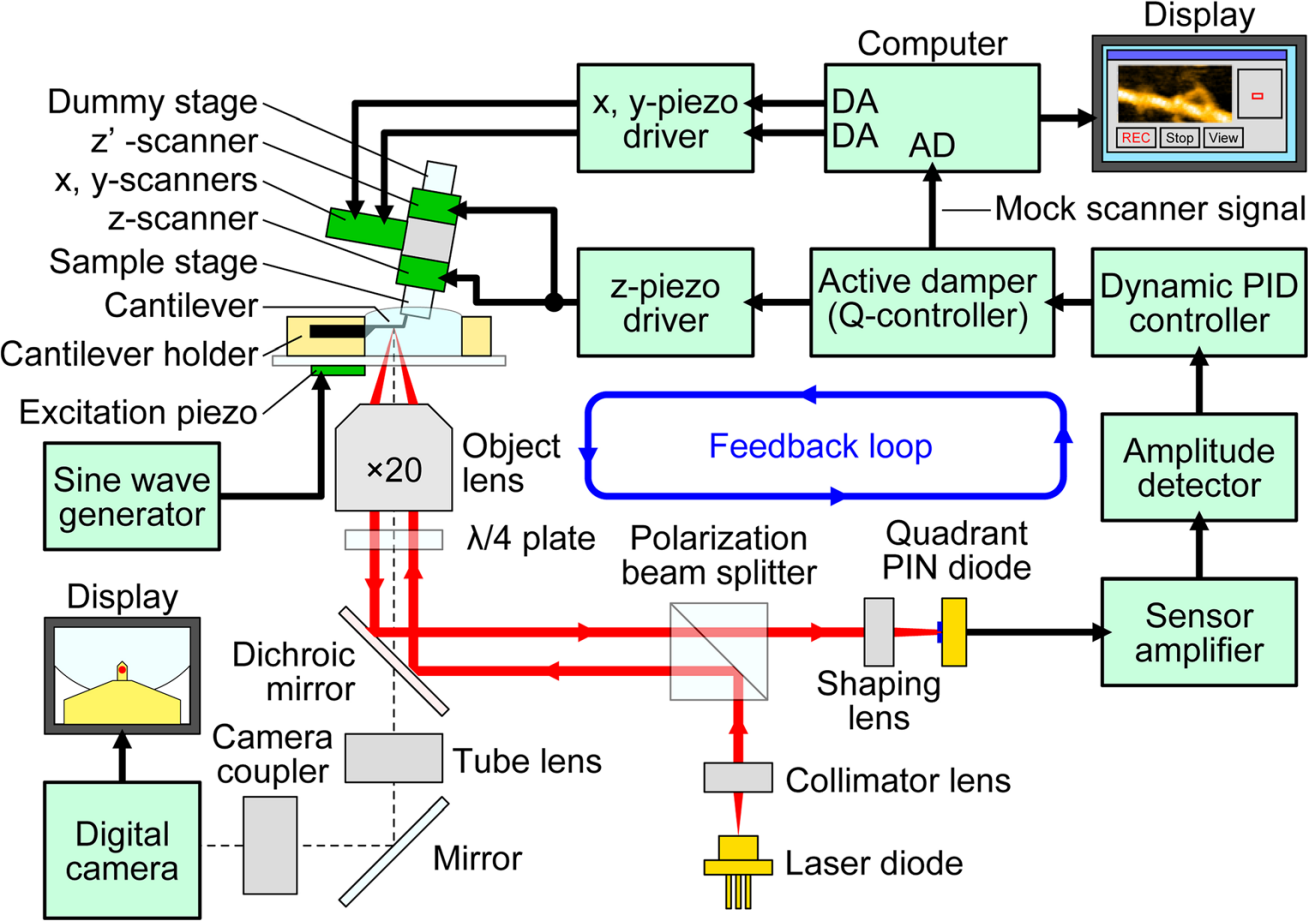
Schematic of HS-AFM system. The system includes an original inverted optical microscope. The objective lens with a long working distance that is a part of the OBD detector is also used to view the cantilever and sample stage via a digital camera. The glass slide, to which the cantilever holder and the liquid cell are attached, is placed on the optical microscope stage. A cantilever chip is held in the holder so that its tip points upward (opposite to the way in conventional AFM). The sample stage, attached to the Z-scanner and facing downward, is placed over the cantilever. An incident laser beam passing through the objective lens is focused onto the small cantilever, and the light reflected back from the cantilever is collected and collimated by the same objective lens and guided to the quadrant-cell Si PIN photodiode. The incident and reflected laser beams are separated using the quarter-wavelength (λ/4) plate and the polarization beam splitter. A cantilever immersed in a buffer solution is oscillated with small amplitude (1–2 nm) by the excitation with an excitation piezo at the first resonant frequency of the cantilever (0.6–1.2 MHz in water). The cantilever oscillation amplitude is maintained constant by feedback control. A counterbalancing method is employed to the Z-scanner to minimize unwanted vibrations; two peazoactuators are attached to the supporting base at its counter sides and simultaneously displaced in the same distance, in the counter directions. The scan signals for the X- and Y-scanner are output from the computer through the DA converters. The output from the active Q-control damper is recorded as the sample height through the AD converter. The active Q-control damper is constructed with an LRC circuit whose transfer function is very similar to that of the Z-scanner. Its electrical output behaves in a way similar to the Z-scanner displacement

HS-AFM has overcome this technical limitation. Biological molecules can be directly observed at sub-molecular spatial and sub-100 ms temporal resolution without disturbing their function. In this section, we briefly summarize the principle of AFM and the current state of HS-AFM performance (Fig. 7). Techniques involved in the HS-AFM instrument are well described elsewhere (Ando et al. 2001; Ando et al. 2008; Ando 2012). See other reviews for protocols of HS-AFM imaging of protein molecules (Uchihashi et al. 2012) and various application studies of HS-AFM (Ando et al. 2014).

### Principles of AFM imaging

In AFM (Binnig et al. 1986), a sharp tip attached to the free end of a cantilever traces the sample to acquire its surface topography while the sample stage is scanned laterally. AFM can observe any object on a substrate at single-nanometer (or higher) resolution under a wide range of environments (vacuum, air, and liquids). Topography images of biological samples can be acquired under physiological buffer conditions, without fixing, staining and labeling the sample (Engel and Müller 2000; Müller and Dufrêne 2008). Among various imaging modes, the tapping mode (Zhong et al. 1993) is most suited for biological samples. In this mode, the cantilever is excited to oscillate in the Z-direction at or near its first resonant frequency. Because of this oscillation, the cantilever tip is brought intermittently into contact with the sample surface, which can eliminate lateral friction force and hence minimize deformation of fragile samples. The reduction of cantilever oscillation amplitude upon tip-sample contact is measured and then the sample stage is finally moved in the Z-direction to restore the amplitude to its set point using a feedback control. This series of operations is repeated many times for different sample surface points during lateral scanning of the sample stage. Consequently, the 3D movement of the sample stage approximately traces the sample surface, and hence, a topography image of the sample can be constructed from the electric signal used to drive the scanner in the Z-direction.

### Current state of HS-AFM

With conventional AFM instruments it takes at least 30 s to capture an image. This slow rate is due mainly to the slow response of the mechanical devices (i.e. cantilevers and Z-scanner), whereas many interesting biological phenomena occur much faster. Various efforts have been made to markedly increase the imaging rate of tapping-mode AFM over the past 20 years. Small cantilevers, fast scanners, and fast amplitude detectors were developed to build the first prototypic instruments (Viani et al. 2000; Ando et al. 2001). The improvement of these devices and creation of new techniques for vibration damping and fast feedback control produced the current HS-AFM (Ando et al. 2008). The highest possible imaging rate is a function of the speed of an AFM instrument as well as the imaging conditions and the sample fragility (Uchihashi et al. 2012; Ando et al. 2013). For 50- to 200-nm scan ranges, sufficient to image biological molecules, HS-AFM can capture an image within 30–80 ms without disturbing their biological function. The spatial resolution of HS-AFM is now comparable with that of conventional slow AFM even with high-bandwidth detection of the cantilever oscillation amplitude and fast scanning of the sample stage (∼2 nm for XY and ∼0.1 nm for Z in the best case). HS-AFM has already visualized dynamic biomolecular phenomena (see reviews in Ando 2012; Ando et al. 2013, 2014) and even dynamic phenomena in live cells (Yamashita et al. 2012; Watanabe et al. 2013).

## Walking M5 filmed by high-speed AFM

### Substrate surface and observation of unidirectional movement

**Fig. 8:**
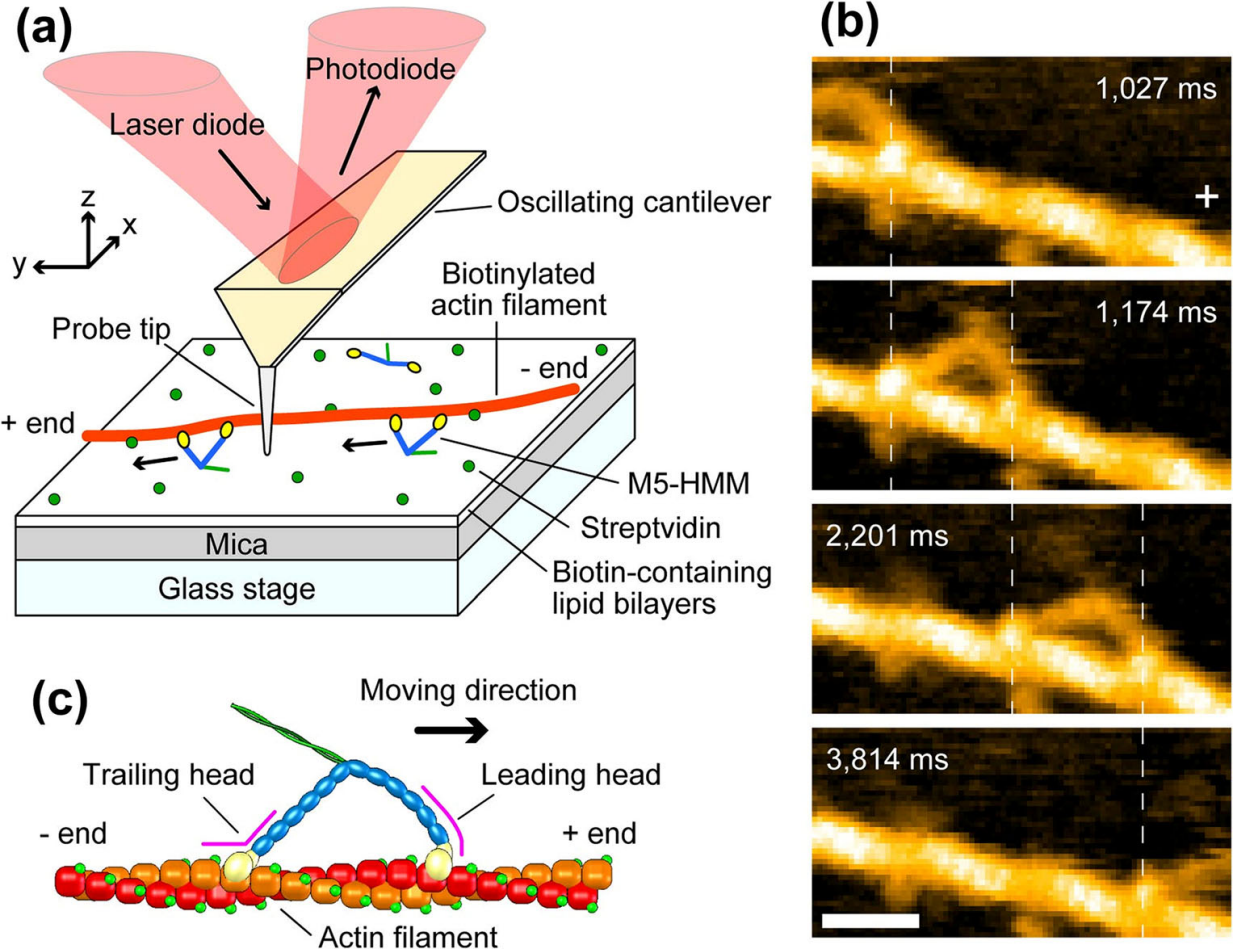
HS-AFM imaging of walking M5-HMM. **a** Schematic showing assay system used for the HS-AFM imaging. **b** Successive HS-AFM images showing unidirectional processive movement of M5-HMM observed in 1 μM ATP. Frame rate, 7 fps; Scan area, 130 × 65 nm^2^ with 80 × 40 pixels; *scale bar* 30 nm. The *vertical dashed lines* show the centers of mass of the motor domains, and the *plus sign* indicates the plus end of actin. **c** Schematic showing two-headed bound M5-HMM to actin. The corresponding AFM movies can be seen at the following web site: http://www.nature.com/nature/journal/v468/n7320/extref/nature09450-s2.mov

M5-HMM was directly imaged walking along actin filaments using HS-AFM (Kodera et al. 2010). Here, partially biotinylated actin filaments were immobilized on a surface where streptavidin was dispersed at low density on mica-supported planar lipid bilayer containing electrically neutral phospholipids and a biotinylated lipid (Fig. 8a). M5-HMM was never bound directly to the lipid bilayer surface and only interacted with the immobilized actin filaments and moved thereon. The moving M5 was frequently oriented perpendicular to the substrate surface so that its characteristic lateral topography was only occasionally observed. Adding a positively charged lipid to the bilayer solved this problem.

In the presence of 1–2 μM ATP, successive AFM images captured at 7 fps clearly showed M5-HMM moving processively with discrete ∼36-nm steps (Fig. 8b). The two-headed bound M5-HMM exhibited unique structural features (Fig. 8c). The junction of the neck with the motor domain appears smooth in the leading head but is always V-shaped in the trailing head because the neck regions emerge from different parts of the motor domain. The short coiled-coil tail was mostly tilted towards the minus end of actin. These structural features, which are totally consistent with an electron microscopic observation (Burgess et al. 2002), can be used to determine the actin polarity when bound M5-HMM is stationary. In addition, the leading head always assumed a straight conformation (slightly curved outwards), which agreed with the straight-neck model proposed for walking M5 (Forkey et al. 2003; Syed et al. 2006; Toprak et al. 2006) but disagreed with the bent-neck model (Walker et al. 2000; Burgess et al. 2002; Snyder et al. 2004; Oke et al. 2010).

The positively charged lipid in the lipid bilayer slightly retarded the translocation velocities. When it was absent, the velocities at various ATP concentrations were similar to those measured by fluorescence microscopy under the same buffer solution condition (Forkey et al. 2003; Baker et al. 2004), indicating negligible effects of the tip-sample interaction on the motor activity. However, molecular behavior during a step could not be resolved as it completed very fast.

### Visualization of stepping behavior

**Fig. 9:**
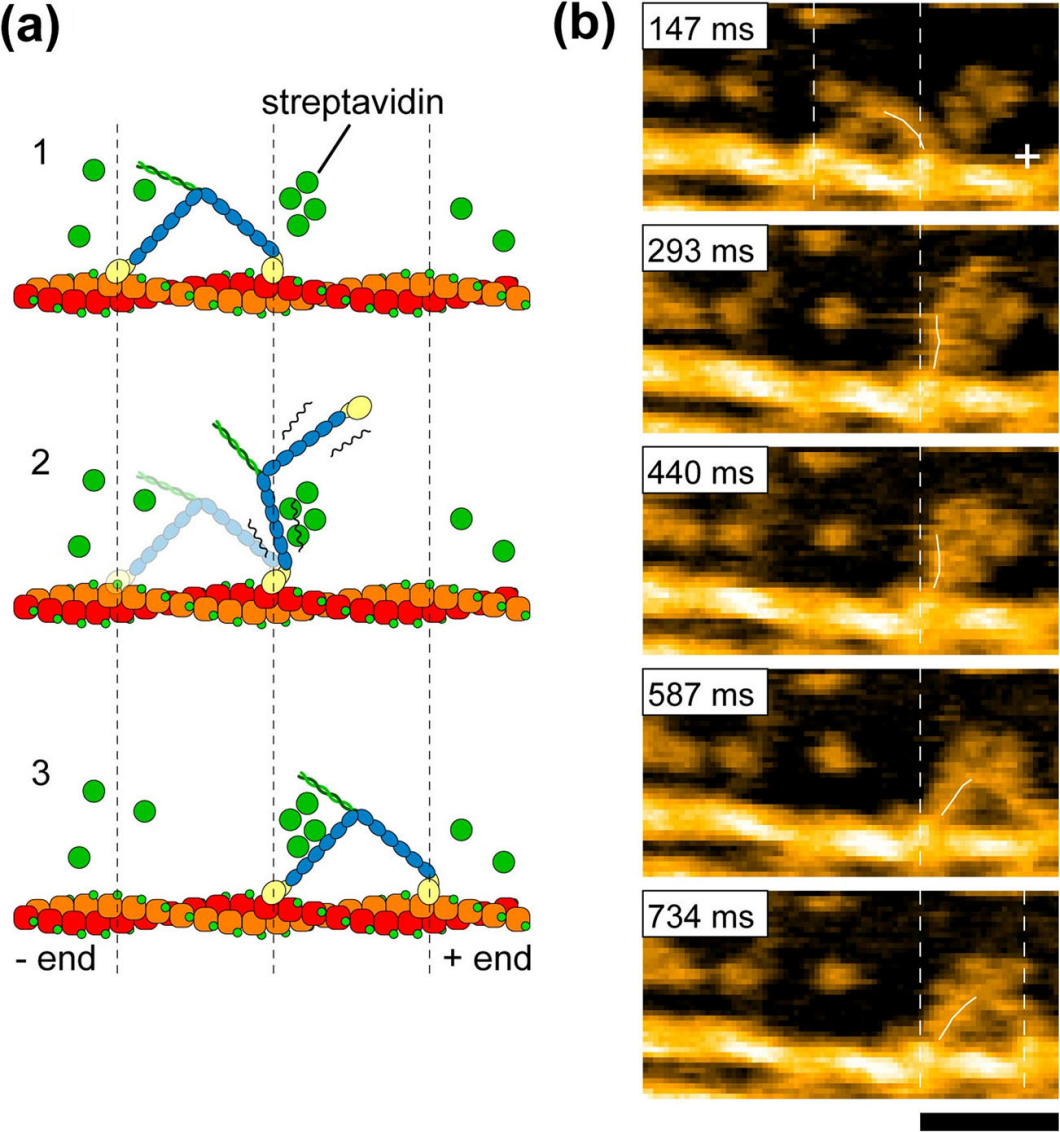
Stepping behavior of M5-HMM visualized by HS-AFM. **a** Schematic explaining the HS-AFM images shown in (**b**). **b** Successive HS-AFM images that resolved the stepping behavior of M5-HMM in 1 μM ATP. Frame rate, 7 fps; scan area, 150 × 75 nm^2^ with 80 × 40 pixels; s*cale bar *50 nm. The swinging lever-arm is highlighted with the *thin white lines*. The *vertical dashed lines* in (**a**) and (**b**) represent the centers of mass of the motor domains, and the *plus sign* indicates the plus end of actin. The corresponding AFM movies can be seen at the following web site: http://www.nature.com/nature/journal/v468/n7320/extref/nature09450-s3.mov

To slow the step, more streptavidin molecules were placed on the lipid bilayer surface as moderate obstacles to the walking of M5-HMM (Fig. 9a), which resolved the stepping process (Fig. 9b). Upon detachment of the trailing head from actin, the leading head appeared to spontaneously rotate from the reverse arrowhead orientation towards the arrowhead orientation. Before completing this rotation, the leading head was halted for a moment by colliding with a streptavidin molecule placed on the way of its natural path. In this halt state, the detached trailing head was displaced forward, positioned most distant from the actin filament (thus, the two heads were aligned nearly straight, pointing opposite directions), and slightly rotated around the neck-neck junction (the second frame in Fig. 9b). Then, the leading head overcame the obstacle and further rotated forwards. Immediately after this the trailing head bound to a forward site of the actin filament to become a new leading head, completing one step. Thus, dynamic processes in the forward step were directly visualized. The observed rotation of the leading head is exactly the swinging lever-arm motion itself initially proposed for the powerstroke of muscle myosin (Huxley 1969). Note that before completing a step the detached trailing head never interacted with actin but passively moved forwards driven by the rotating leading head. This ruled out some models of M5 motility such as the ‘inchworm’-like model considered for kinesin (Hua et al. 2002) and the “biased diffusion” model proposed for single-headed myosin II (Kitamura et al. 1999) and single-headed M5 (Okada et al. 2007). M5 strictly followed the hand-over-hand mechanism. In contrast, the other processive myosins VI and X, which function not only as a cargo transporter but also as a structural anchor in cells, were reported to move irregularly, namely inchworm-like stepping, backward stepping, and forward hand-over-hand stepping (Yildiz et al. 2004; Sun et al. 2007; Nishikawa et al. 2010; Ricca and Rock 2010; Sun et al. 2010).

### Foot stomp and unwinding of coiled-coil tail

**Fig. 10:**
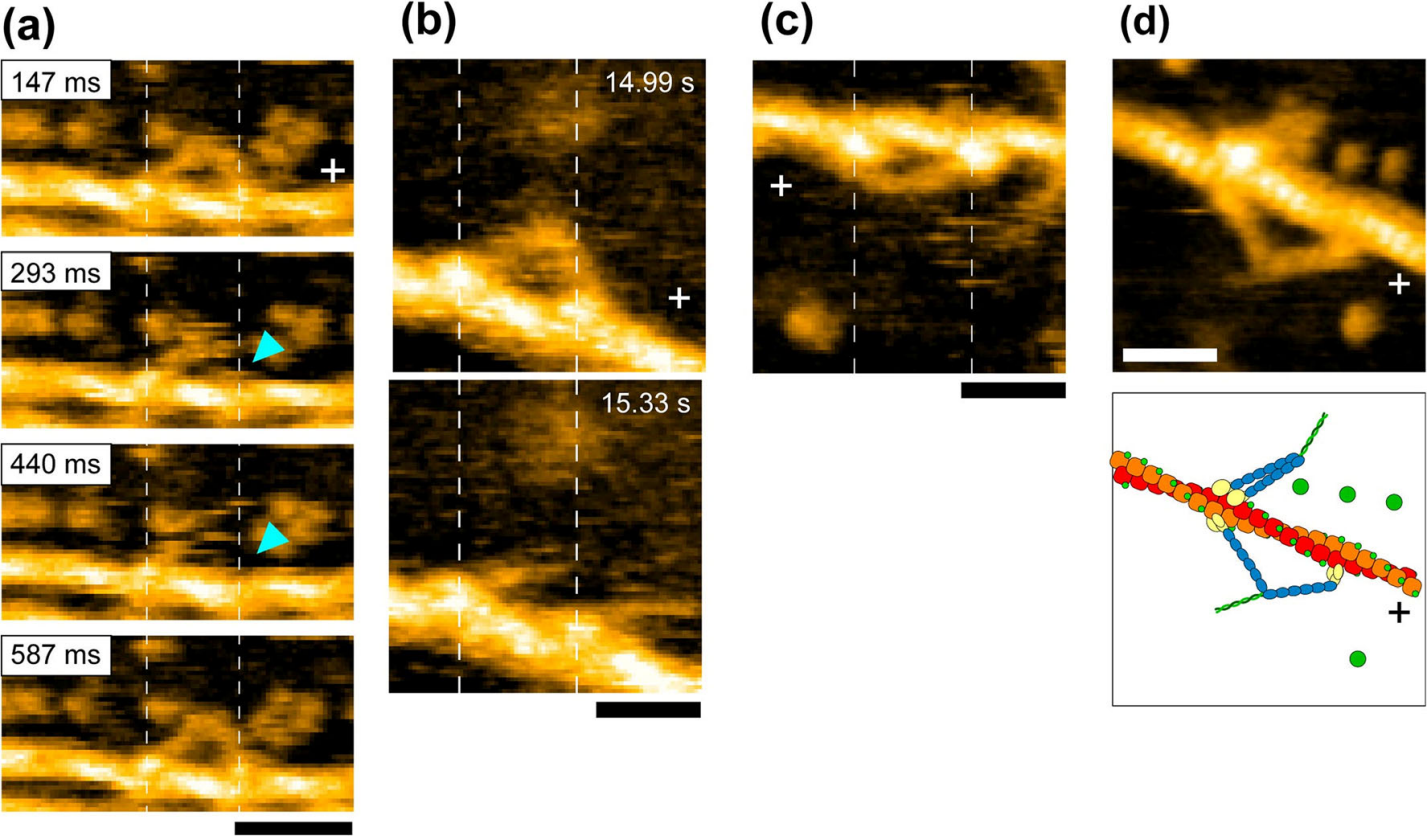
Unique molecular behaviors of M5-HMM visualized by HS-AFM. **a** Successive HS-AFM images showing a foot stomp event that occurred at the leading-head (ATP, 1 μM; frame rate, 7 fps; scan area, 150 × 75 nm^2^ with 80 × 40 pixels; *scale bar* 50 nm). The leading-head detachment is marked with the *light-blue arrowheads*. The corresponding AFM movie can be seen at the following website: http://www.nature.com/nature/journal/v468/n7320/extref/nature09450-s3.mov **b** HS-AFM images before (*upper panel*) and after (*lower panel*) unwinding of coiled-coil tail observed in 50 μM ADP. The corresponding AFM movie can be seen at the following website: http://www.nature.com/nature/journal/v468/n7320/extref/nature09450-s5.mov **c** HS-AFM image showing the leading head with a sharply bent conformation observed in the nucleotide-free condition. The corresponding AFM movie can be seen at the following website: http://www.nature.com/nature/journal/v468/n7320/extref/nature09450-s6.mov **d** HS-AFM image showing nucleotide-free M5-HMM with heads bound to adjacent actin subunits (*upper panel*) and its illustration (*lower panel*). Imaging conditions for (**b**), (**c**) and (**d**) are as followings: frame rate, 3 fps; scan area, 100 × 100 nm^2^ with 80 × 80 pixels; *scale bar* 30 nm. The *vertical dashed lines* represent the centers of mass of the motor domains, and the *plus signs* indicate the plus ends of actin

Interestingly, in the two-headed bound state during the dwell, the motor domain of the leading head frequently exhibited brief dissociation and reassociation on the same actin filament, while the molecule remained at approximately the same position on the filament (Fig. 10a). Similarly, the motor domain of the trailing head exhibited a brief translocation by ∼±5 nm along the actin filament. We termed these behaviors “foot stomp”. The foot stomp was more frequently observed at the leading head than at the trailing head (by a ratio of approximately 3:1). Although not well documented, a foot-stomp-like behavior was previously suggested in fluorescence microscopy observations of walking M5 molecules (Syed et al. 2006; Shiroguchi and Kinosita 2007). More recently, the foot stomp was further confirmed by the observation of walking M5 by high-speed single-molecule polarized fluorescence microscopy (Beausang et al. 2013). Thus, the foot stomp is an inherent behavior of this motor.

The foot stomp at the leading head raises an important question as to the chemo-mechanical coupling in M5. The briefly detached leading head does not carry bound Pi because Pi has already been released from the leading head following its initial attachment to actin (De La Cruz et al. 1999; Olivares et al. 2006). Nevertheless, the detached leading head with its bound ADP reattached to actin while still in the reverse arrowhead orientation. It then swung forward following trailing head detachment. This fact was important because it indicated that tension generation for forward movement can occur without transitioning through the ADP–Pi bound state, i.e. it can occur directly in the ADP–bound state. Thus, the tension generation for forward movement does not seem to require chemical energy be supplied by ATP hydrolysis.

The asymmetry of foot stomp frequency between the two heads suggested their actin-affinity difference, consistent with a biochemically measured result (Olivares et al. 2006). The leading head binds to actin in the unnatural orientation (i.e. reverse arrowhead orientation), and hence, pays an energy cost by distorting the neck conformation, resulting in a lower affinity for actin. The distortion of the leading head is likely to be the source for tension generation for forward movement. This was reinforced by the following observation; when the two-heads were bound to actin in 1 mM ADP, the short coiled-coil tail was sometimes unwound, immediately after which the monomerized leading head rotated towards the arrowhead orientation, similar to the swinging lever-arm motion observed when M5-HMM was walking forwards (Fig. 10b).

### Flexibility of neck-motor domain junction

Under nucleotide-free conditions, the leading head frequently exhibited a sharply bent conformation (Fig. 10c) that was never observed in the presence of ATP or in the presence of 1 mM ADP. This bent conformation suggested that the neck-motor domain junction is less flexible in the nucleotide-free head than in the nucleotide-bound head. To examine this issue, the arrowhead orientation angle of single-headed M5 relative to the actin filament was measured under the nucleotide-free and ADP-bound conditions. In nucleotide-free solution, the angle was ∼34°, whereas in ADP the angle was distributed widely, peaked at ∼29° and ∼51° in the proportion of approximately 6 to 1 (supplementary data in Kodera et al. 2010), which may be relevant to the two different ADP-bound states in equilibrium as reported for myosin II (Geeves 1989; Geeves and Holmes 1999), M5 (Robblee et al. 2005; Hannemann et al. 2005; Oguchi et al. 2008; Jacobs et al. 2011), and myosin VI (Robblee et al. 2005). Therefore, ADP binding to the head makes the hinge of the neck–motor domain junction flexible. Because of the rigid hinge of the nucleotide-free head, the leading neck in the reverse arrowhead orientation tends to be sharply bent to release the large strain accumulated therein. This rigid hinge was also supported by the observation that both heads are rarely bound to adjacent actin subunits but only in the nucleotide-free condition (Fig. 10d). In the electron micrographs of actomyosin II and actomyosin VI complexes, this conformation was frequently seen, suggesting they have rigid hinges (Craig et al. 1980; Nishikawa et al. 2002).

The flexibility of the neck-motor domain junction observed for the actin-bound M5 head with ADP does not seem sufficient to account for the two-headed binding in ADP because the leading head binding in the reverse-arrowhead orientation requires the junction to bend by at least ∼60°, even when the contribution of the neck domain’s flexibility is considered. This apparently restricted flexibility suggests that the actin-unbound head with ADP is more flexible than the actin-bound one. In the other words, actin binding may make the neck-motor domain junction less flexible. This plausible flexibility change seems an excellent strategy for facilitating both actin binding and the generation of enough tension to execute a powerstroke. Very recently, such an actin-binding effect on the flexibility was proposed for muscle myosin (Billington et al. 2014).

### Asymmetry of ADP dissociation rate

The straight and sharply bent conformations of the leading head depend on the presence or absence of bound ADP, respectively, meaning that one can judge whether or not the leading head contains ADP by just looking at its conformation. From the proportion and lifetime of the straight conformation as a function of ADP concentration, the rate constants of ADP binding/dissociation kinetics at the leading head were estimated. The ADP dissociation rate constant was 0.1 s^−1^, i.e. one ADP is released from the leading head every 10 s, on average. However, M5 walks many steps during 10 s. Thus, from the structural point of view, it was confirmed that the sequential events of ADP release, the subsequent ATP binding and the resulting head dissociation take place solely at the trailing head, which is the basis for the processive hand-over-hand movement (Rosenfeld and Sweeney 2004; Purcell et al. 2005; Veigel et al. 2005; Forgacs et al. 2008; Oguchi et al. 2008; Sakamoto et al. 2008).

## New questions

The swinging lever-arm hypothesis is no longer a hypothesis. HS-AFM visualized its occurrence with irrefutable clearness. However, from HS-AFM observations of M5-HMM interacting with actin, questions were raised about the chemo-mechanical coupling in this motor as well as myosin motors in general. The prevailing coupling mechanism had been modeled in the context of swinging lever-arm motion (Fig. 3). In this model, the structural states are putatively tightly coupled to the nucleotide states of the motor domain (Fig. 3). In the M–ADP–Pi state, the head takes the pre-powerstroke conformation caused by recovery stroke of the lever-arm, which facilitates binding of the head to actin in the reverse arrowhead orientation. Coupled to Pi release from the actin-bound head, the intramolecular tension is considered to be generated just once by the pre-powerstroke to post-powerstroke transition. However, the observation with HS-AFM of walking M5-HMM showed that the tension responsible for the lever-arm swing can be generated directly by the ADP-bound leading head (after foot stomping). In addition, it was shown that two-headed bound M5-HMM with ADP alone can generate sufficient tension to cause occasional unwinding of the short coiled-coil tail, after which the lever-arm swings. Thus, the recovery stroke or the pre-powerstroke conformation thought to occur uniquely in the ADP–Pi-bound head does not seem necessary for its binding to actin and lever-arm swing. More seriously, the chemical energy liberated by ATP hydrolysis does not seem to be used for the processes of recovery stroke, tension generation, and lever-arm swing, suggesting that M5 would step forward without chemical energy input once the trailing head detaches from actin.

This new idea of “no chemical energy usage” in these processes would, however, inevitably lead us to encounter a “perpetuum mobile problem”. Even in ADP or in the nucleotide-free condition, foot stomp occurs at both leading and trailing heads of two-headed bound M5-HMM, with a higher frequency in the leading head than in the trailing head. When the trailing head detaches from actin, the molecule takes a forward step by the spontaneous swing of the leading head. On the other hand, when the leading head detaches from actin, the molecule cannot take a backward step because the trailing head is bound to actin in a stable orientation (i.e. the arrowhead orientation). Thus, using only thermal energy, M5 would step forward many times (albeit very slowly), even with less frequent occurrence of foot stomps at the trailing head. This is a forbidden perpetuum motion. However, in this *gedankenexperiment*, positional fluctuations of the single-headed bound molecule due to the flexible neck-motor domain junction are not considered. Taking this into account, we can expect a backward step after detachment of the leading head from actin, albeit with a low probability. The ratio of foot stomp occurrence between the leading and trailing heads was observed to be 4:1 in the presence of 1 mM ADP. However, the foot stomp events at the trailing head mostly occurred as a brief ±5-nm translocation. Therefore, when this brief translocation is omitted and only the events of brief detachment from actin are counted as foot stomp, the ratio of foot stomp occurrence between the two heads becomes much larger. Thus, the principle of detailed balance holds between the forward step after trailing head detachment and the backward step after leading head detachment, resulting in no net movement of the molecule.

From numerous X-ray crystallography and electron microscopy studies, there is no doubt that myosin changes its conformation upon binding nucleotides and takes a pre-powerstroke-like conformation in the ADP–Pi-bound state (Fisher et al. 1995; Smith and Rayment 1996; Dominguez et al. 1998; Houdusse et al. 2000; Burgess et al. 2002; Coureux et al. 2004; Volkmann et al. 2005). These static structures have been used to construct the prevailing model of the chemo-mechanical coupling (Fig. 3) assuming there is a tight one-to-one relationship between the chemical and conformational states. In reality, the relationship is not so tight. Myosin takes two (or multiple) conformations even under a given nucleotide condition. They can go back and forth but the dynamic equilibrium is biased to one side depending on the nucleotide condition. The HS-AFM observations of M5-HMM indicates that structural fluctuations indeed exist and therefore two-headed binding occurs even in the ADP-bound and nucleotide free conditions, consistent with other studies (Walker et al. 2000; Rosenfeld and Sweeney 2004; Olivares et al. 2006).

The flexibility of myosin head conformation raises a question whether the chemical energy of ATP hydrolysis is really used to change the conformation. In the prevailing view, the myosin head with a strained (tension-generating) conformation in the pre-powerstroke state is achieved using the chemical energy of ATP, which is finally released by the swinging lever arm motion. However, the pre-powerstroke and post-powerstroke conformations are in dynamic equilibrium with no marked energy barrier. Thus, one can conclude as follows: although the conformational change of myosin head caused by recovery stroke is an effective strategy for facilitating its binding to actin in the reverse arrowhead orientation, the energy required for producing a pre-powerstroke conformation and the tension-generating state is much less than that to be liberated by ATP hydrolysis. Therefore, the conformational change can occur thermally without chemical energy input. At first glance this new view seems inconsistent with the recent report (Shiroguchi et al. 2011) in which the energy barrier between the pre-powerstroke state conformation (formed immediately after ATP addition) and post-powerstroke conformation (formed in the absence of nucleotides) was estimated to be 5.2 *k*_B_ *T* (*k*_B_, Bolzmann constant; *T*, room temperature in Kelvin). For this estimation, they monitored the angular distributions of a large bead duplex attached to the motor domain of a single-headed M5 construct, while the molecule was immobilized to a surface of a 50-nm bead by the neck region. This estimated energy barrier seems too large to be achieved thermally. However, the angle distribution measurement did not include the contribution of the flexible neck region to the angle fluctuations, because the neck region was attached to a surface. If it was included, the energy barrier could be smaller. Moreover, the observed angular distributions only reflect projections in the image plane. Therefore, the distributions are underestimated and hence the energy barrier is overestimated. In fact, from solution kinetics measurements of muscle myosin ATPase reaction, the free energy change in the recovery stroke-involved transition from M–ATP to M–ADP–Pi is estimated to be 2 *k*_B_T (Howard 2001). Moreover, the attachment of a large bead duplex to the head would significantly impede the otherwise frequent thermal transitions between the pre-powerstroke and post-powerstroke states.

Let us perform another *gedankenexperiment* in the presence of ADP alone. Suppose each head of M5 is labeled with a chromophore. Upon light absorption, the chromophore on the bound head will alter its conformation so that it dissociates from actin. When a light pulse is illuminated only at the trailing head of the bound M5, it spontaneously steps forward after detachment of the trailing head. By repeated light illumination only at the trailing head, the molecule would move for a long distance unidirectionally.

It seems plausible that a large fraction of the chemical energy liberated by ATP hydrolysis is used elsewhere and could be used to detach the actin-bound nucleotide-free head from actin. How much energy is required? As mentioned in “Single-molecule measurements”, the unbinding (rupture) force was previously measured to be ∼15 pN by AFM (Nakajima et al. 1997). The rupture distance was also estimated to be 1.7–2.5 nm in this study. This distance is unusually long compared to 0.23 nm for antigen–antibody (Hinterdorfer et al. 1996), 0.15–0.3 nm for streptavidin–biotin (Yuan et al. 2000), and 0.05–0.3 nm for α-actinin–actin (Miyata et al. 1996). However, it is consistent with the fact that actin–myosin interfaces contain several bonds formed by flexible loops (Milligan 1996; Kabsch et al. 1990). From the values of rupture force and rupture distance, the energy required to rupture the actin–myosin rigor bond is 6–9 *k*_B_ *T*, which is 30–45 % of the chemical energy of ATP hydrolysis (∼20 *k*_B_ *T*). This mechanically estimated value is approximately consistent with the values of 9 and 10 *k*_B_ *T* estimated biochemically for the free energy changes in the dissociation A–M → A + M and the ATP-induced dissociation A–M + T → A + M–T, respectively (Howard 2001). One may argue against this statement. For instance, ATP is not yet hydrolyzed in the actin–myosin dissociation step, and hence the energy of ATP is not yet liberated in this step. However, the account balance of energy should be considered for the whole cycle of the ATPase reaction. Therefore, unlike chemical cleavage reactions, in the enzyme-catalyzed reaction cycle the ATP-binding energy can be used as a large part of the energy liberated by ATP hydrolysis. The role of the sequential ATPase reaction process after ATP binding and head dissociation from actin would lie in ensuring the unidirectional progression of the mechanical process. For this assurance, only a small fraction of ATP energy, comparable to thermal energy, is sufficient.

## Conclusions

We have shown how our understanding of the myosin motor mechanism has advanced with increasing level of directness of measurement. When the level of directness is low, arguments can continue without resolution. For example, in the efforts to provide evidence for or against the swinging lever-arm hypothesis, experimental data on the structure and dynamic behavior of myosin were obtained separately. Detailed but static structural data have often given the impression that there is a tight relationship between structural and chemical states. The single-molecule biophysical studies of the dynamic behavior of myosin, on the other hand, have revealed a subset of dynamic molecular events that depends on where the optical probe is placed, and hence other subsets are missed. Therefore, constructing models for the myosin motor mechanism on this basis is reminiscent of a jigsaw puzzle with missing pieces that have to be filled by speculation and hypotheses. Directly observing the structural dynamics and dynamic processes of M5-HMM by HS-AFM has significantly reduced the missing parts and revealed a picture that appears similar to but is largely different from that envisaged previously. Surprisingly, it revealed that generation of tension responsible for forward movement and the following lever-arm swing can occur without through the ADP–Pi bound state and therefore the mechanical events do not require the input of chemical energy. Thus, the chemical energy of ATP is mainly used for the detachment of the trailing head from actin. Once detached, the forward swing of the leading head and the resulting forward step of the molecule occur spontaneously without chemical energy input. Of course, this issue of energy usage by M5 should be further corroborated in more direct ways. Moreover, this new principle of energy usage needs to be confirmed for other myosin motors as well as for other mechano-enzymes. This important task remains to be accomplished.
